# Isolation and Structural Elucidation of Compounds from *Pleiocarpa bicarpellata* and Their In Vitro Antiprotozoal Activity

**DOI:** 10.3390/molecules27072200

**Published:** 2022-03-28

**Authors:** Ozlem Sevik Kilicaslan, Sylvian Cretton, Luis Quirós-Guerrero, Merveilles A. Bella, Marcel Kaiser, Pascal Mäser, Joseph T. Ndongo, Muriel Cuendet

**Affiliations:** 1 School of Pharmaceutical Sciences, University of Geneva, 1211 Geneva, Switzerland; ozlem.sevik@unige.ch (O.S.K.); sylvian.cretton@unige.ch (S.C.); luis.guerrero@unige.ch (L.Q.-G.); 2 Institute of Pharmaceutical Sciences of Western Switzerland, University of Geneva, 1211 Geneva, Switzerland; 3 Department of Chemistry, Higher Teacher Training College, University of Yaoundé 1, Yaoundé P.O. Box 47, Cameroon; bella_aurore@yahoo.fr (M.A.B.); thierry.ndongo@ens.cm (J.T.N.); 4 Swiss Tropical and Public Health Institute, 4002 Basel, Switzerland; marcel.kaiser@swisstph.ch (M.K.); pascal.maeser@swisstph.ch (P.M.); 5 University of Basel, 4003 Basel, Switzerland

**Keywords:** *Pleiocarpa*, dereplication, alkaloids, antiprotozoal activity, malaria

## Abstract

Species of the genus *Pleiocarpa* are used in traditional medicine against fever and malaria. The present study focuses on the isolation and identification of bioactive compounds from *P. bicarpellata* extracts, and the evaluation of their antiprotozoal activity. Fractionation and isolation combined to LC-HRMS/MS-based dereplication provided 16 compounds: seven indole alkaloids, four indoline alkaloids, two secoiridoid glycosides, two iridoid glycosides, and one phenolic glucoside. One of the quaternary indole alkaloids (**7**) and one indoline alkaloid (**15**) have never been reported before. Their structures were elucidated by analysis of spectroscopic data, including 1D and 2D NMR experiments, UV, IR, and HRESIMS data. The absolute configurations were determined by comparison of the experimental and calculated ECD data. The extracts and isolated compounds were evaluated for their antiprotozoal activity towards *Trypanosoma brucei rhodesiense*, *Trypanosoma cruzi*, *Leishmania donovani*, and *Plasmodium falciparum,* as well as for their cytotoxicity against rat skeletal myoblast L6 cells. The dichloromethane/methanol (1:1) root extract showed strong activity against *P. falciparum* (IC_50_ value of 3.5 µg/mL). Among the compounds isolated, tubotaiwine (**13**) displayed the most significant antiplasmodial activity with an IC_50_ value of 8.5 µM and a selectivity index of 23.4. Therefore, *P. bicarpallata* extract can be considered as a source of indole alkaloids with antiplasmodial activity.

## 1. Introduction

Human protozoal diseases cause significant morbidity and mortality. Malaria is one of the most widespread and severe among such diseases. According to a recent report from the World Health Organization (WHO), almost half of the world’s population is at risk of malaria, mostly in Africa where the disease is endemic. In 2021, 241 million malaria cases were estimated, and 627,000 people died from it, mainly children under the age of 5 and pregnant women [1].

Since 2006, WHO recommends the use of artemisinin-based combination therapies (ACTs) as the first-line treatment to reduce the risk of resistance. ACTs combine an artemisinin derivative with a longer half-life anti-malarial drug to improve efficacy and reduce the risk of emergence of new resistant strains. Unfortunately, resistance to ACT has recently been reported in several southeast Asian countries [2]. Hence, there is an urgent global need to search for new, safe, more effective and affordable antimalarial drugs.

*Pleiocarpa* is a genus belonging to the Apocynaceae family, native to tropical Africa and distributed from Senegal to Tanzania and Zimbabwe. This genus consists of six accepted species. Medicinal plants from this chemically under-investigated genus are well known for their use in the traditional treatment of fever, pain, and stomachache [3]. One of them, *P. mutica*, is used to treat fever and malaria in Ghana. A methanolic extract from the roots showed significant activity with an IC_50_ value of 16.7 µg/mL against *P. falciparum* [4]. Antihypertensive and nematocidal activities have also been reported in previous studies [5]. Various secondary metabolites such as indole, bisindole alkaloids, and triterpenoids have been reported from *Pleiocarpa* species [3]. The occurrence of indole alkaloids appears to be important in the family and they were proposed as a novel chemical class of antiplasmodial agents [6]. To the best of our knowledge, the chemical and biological constituents of *P. bicarpellata* Staph. have not been reported so far.

As part of our ongoing research on the discovery of new antiparasitic compounds from African plants, the main purpose of this study was to investigate an extract of *P. bicarpellata*. The dichloromethane/methanol (1:1) extract from the roots of *P. bicarpellata* showed a strong antiplasmodial activity with an IC_50_ value of 3.5 µg/mL against *P. falciparum*. This study led to the isolation of sixteen compounds, including seven indole alkaloids, four indoline alkaloids, two secoiridoid glycosides, two iridoid glycosides, and one phenolic glucoside. Among these, one quaternary indole alkaloid and one indoline alkaloid are described here for the first time. The antiprotozoal activities of all the isolated compounds were evaluated, and these results are detailed herein.

## 2. Results and Discussion

### 2.1. In Vitro Antiprotozoal Activity of the Extracts

The in vitro antiprotozoal activity (against *T. b. rhodesiense*, *T. cruzi*, *L. donovani*, and *P. falciparum)* and cytotoxicity against rat myoblast L6 cells of the *P. bicarpellata* extracts were evaluated. The extracts were considered inactive when their IC_50_ values were >50 µg/mL against the parasites. The dichloromethane/methanol (1:1) extract from the roots demonstrated an activity against the NF54 strain of *P. falciparum* with an IC_50_ value of 3.5 µg/mL without cytotoxicity towards mammalian L6 cells at a concentration of 100 µg/mL (Table 1). Species of the genus *Pleiocarpa* are already known to be a rich source of important bioactive compounds such as indole, bisindole alkaloids, and triterpenoids. Indole alkaloids are reported to possess various biological and pharmacological activities such as antihistamine [7], antifungal [8], antimicrobial [9], antioxidant [10], plant growth regulator [11], anti-HIV [12], anticonvulsant [13], anti-inflammatory, cancer chemo-preventive [14], and analgesic [15] properties. Alkaloids isolated from the roots of *P. mutica* showed potent activity against *P. falciparum* and are responsible for the strong antiplasmodial activity of the extract. Despite the number of published studies on various *Pleiocarpa* species, *P. bicarpellata* has not been extensively studied from a chemical and pharmacological point of view.

### 2.2. LC-ESI-MS/MS Profiling

The extracts were analyzed by ultrahigh-performance liquid chromatography-high-resolution mass spectrometry (UHPLC-HRMS/MS) and the data were used to generate a molecular network. Data generated in the positive mode demonstrated a better ionization efficiency, and therefore, were selected for the annotation of metabolites. MzMine [16] was used to process the data and Cytoscape [17] to visualize the molecular network. Various dereplication tools were used: Global Natural Product Social (GNPS) molecular network platform [18] and In-Silico Database of Natural Products (ISDB) [19]. The chemical profiling of the extracts suggested the presence of classes such as alkaloids and iridoid monoterpenes (Figure S1, Supplementary Materials). The same molecules were putatively identified in the root and stem extracts of *P. bicarpellata*. Moreover, a fragment ion at *m*/*z* 144 [C_10_H_10_N]^+^ indicative of monoterpene indole alkaloids [20] was observed for several compounds. According to the *m*/*z* value and MS/MS fragment ion, a node of the cluster at *m*/*z* 325.191 was attributed to tubotaiwine (**13**). It was assumed that the absence of annotation of the other isomers at *m*/*z* 325.1928 when dereplicated against all the databases indicated possible new analogues. Kopsinine (**2**) was observed and annotated for the node at *m*/*z* 339.2069 in the same cluster than tubotaiwine (**13**). Other indole alkaloids with an *m*/*z* at 517.2198, 313.1915 and 327.1069, which have never been described in the genus *Pleiocarpa* and/or for antiparasitic activities, were putatively identified. Therefore, these compounds were targeted for isolation.

### 2.3. Structural Elucidation

**Figure 1:**
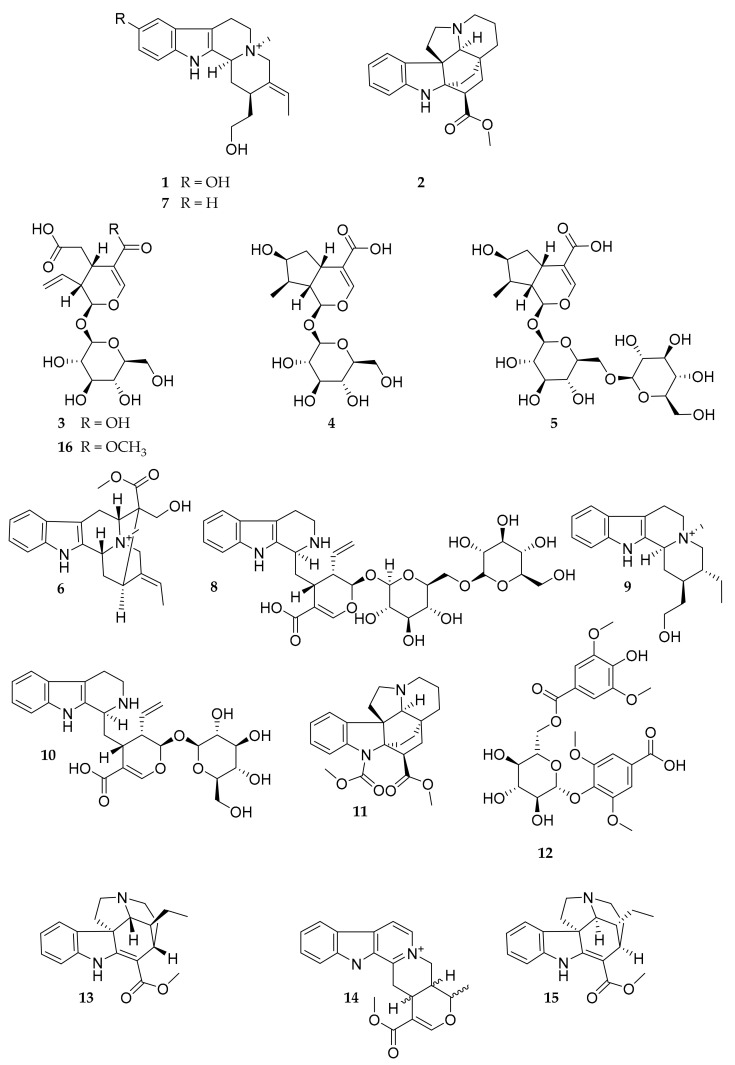
Structures of the isolated compounds from *Pleiocarpa bicarpellata*.

The study of the root and stem extracts of *P. bicarpellata* afforded sixteen compounds. Compounds were isolated using different chromatographic methods. The known compounds were identified as 10-hydroxy-N_b_-methyl-corynantheol (**1**) [21], kopsinine (**2**) [22], secologanoside (**3**) [23], loganic acid (**4**) [24], loganic acid 6′-*O*-β-d-glucopyranosyl (**5**) [25], macusine A (**6**) [26], strictosidinic acid 6′-*O*-β-d-glucopyranoside (**8**) [27], dihydro-N_b_-methyl-corynantheol (**9**) [28], strictosidinic acid (**10**) [29], pleiocarpine (**11**) [30], 4-({6-*O*-[(4-hydroxy-3,5-dimethoxyphenyl)carbonyl]-β-glucopyranosyl}oxy)-3,5-dimethoxybenzoic acid (**12**) [31], tubotaiwine (**13**) [32], serpentine (**14**) [33], and secoxyloganin (**16**) [23] by comparison of their NMR and MS data with those published previously (Figure 1). To our knowledge, nine compounds (**3**–**6**, **8**, **10**, **12, 14** and **16**) have not yet been reported in the genus *Pleiocarpa*. Morever, one quaternary indole alkaloid (**7**) and one indoline alkaloid (**15**) have never been reported before. The elucidation of their structure is described below.

Compound **7** was isolated as a brown oil, with the molecular formula C_20_H_27_N_2_O^+^, which was deduced from the HRESIMS ion peak at *m*/*z* 311.2114 ([M])^+^ (calcd for C_20_H_27_N_2_O^+^, 311.2118) (Figure S4). The IR spectrum showed the absorptions ascribed to an alkene (3308 cm^−1^), a quaternary N-methyl group (2943 and 2831 cm^−1^), and an alcohol group (1023 cm^−1^) (Figure S2). The DEPTQ NMR spectrum of compound **7** (Table 2) indicated the presence of 20 carbons, including one methyl group, six methylenes, seven methines, five quaternary carbons, and a N-methyl group at δ_C_ 48.8 (Figure S6).

**Figure 2:**
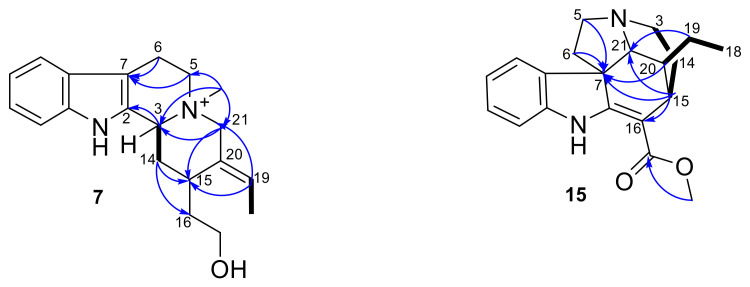
Key COSY (black bold line) and HMBC (blue arrows) correlations of compounds **7** and **15**.

The HMBC correlations from N-CH_3_ (δ_H_ 3.18) to C-3 (δ_C_ 65.9), C-5 (δ_C_ 59.5), and C-21 (δ_C_ 63.8) confirmed the attachment of the methyl group to the nitrogen atom (Figure 2). The two sp2 carbons at δ 128.5 (C-2) and 105.4 (C-7) revealed the Δ2(7) double bond of the indole moiety. Another double bond was evidenced by the HMBC correlations between the olefinic proton H-19 (δ_H_ 5.97) and C-18 (δ_C_ 13.4), C-20 (δ_C_ 129.0), and C-21 (δ_C_ 63.8), which suggested the attachment of H-19 at C-20 (Figure S9). Overall, the ^1^H and DEPTQ NMR spectra of compound **7** revealed great similarities to those of compound **9**, except for the presence of an alkene signal at δ_H_ 6.11 (q, *J* = 5.97, H-19). 

**Figure 3:**
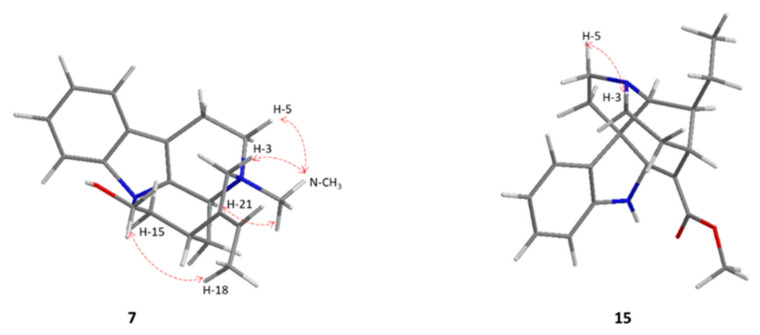
3D structures and key ROEs correlations of compounds **7** and **15**.

**Figure 4:**
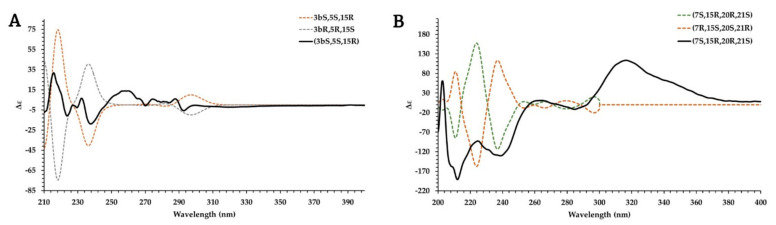
Experimental and TDDFT simulated ECD spectra of compounds **7** (**A**) and **15** (**B**).

A ROESY experiment showed a ROE correlation between N-CH_3_ (δ_H_ 3.18) with H-21, H-5, and H-3 (Figure 3 and Figure S10). This suggested a *cis*-isomerism between the N-CH_3_ and H-3. To establish the absolute configuration of the carbons N-CH_3_, C-3, and C-15, the ECD spectrum of **7** was measured and compared with calculated ECD data. The experimental spectrum (Figure 4) showed negative Cotton effects (CE) at 214, 232, and 255 nm and positive CEs at 222 and 235 nm that matched well with the calculated ECD curve for *3bS,5S,15R*. Accordingly, compound **7** was identified as (*3bS,5S,15R,E*)-20-ethylidene-15-(15-hydroxyethyl)-5-methyl-3,3b,5,6,14,16,17,21-octahydro-1*H*-indolo[15,20-*a*]quinolizin-5-ium and named N_b_-methyl-corynantheol.

Compound **15** was isolated as a colorless amorphous solid with the molecular formula C_20_H_24_N_2_O_2_, which was indicated by the HRESIMS protonated ion peak at *m*/*z* 325.1901 ([M + H])^+^ (calcd for C_20_H_24_N_2_O_2_, 325.1911) (Figure S12). The ^1^H and DEPTQ NMR spectra of compound **15** (Table 2) are similar to those of tubotaiwine (**13**). Indeed, the ^1^H-NMR spectrum of compound **15** displayed signals attributed to a methoxy group (δ_H_ 3.75, 3H, s), an indole NH (δ_H_ 8.57, 1H, br s), and four aromatic protons of a 1,2-disubstituted benzene moiety at δ_H_ 7.34 (d, *J* = 7.5 Hz, H-9), 7.18 (td, *J =* 7.7 Hz, H-11), 6.98 (d, *J =* 7.9 Hz, H-12), and 6.93 (td, *J* = 7.4 Hz, H-10) (Figure S13). The DEPTQ NMR spectrum of **15** indicated the presence of 20 carbons, which were attributed, with the assistance of the HSQC spectrum, to one methyl at δ_C_ 11.4, five methylenes (δ_C_ 24.3, 27.9, 42.9, 46.6, 53.5), seven methines (δ_C_ 31.2, 41.3, 66.6, 111.7, 120.8, 122.6, 129.6), six quaternary carbons (δ_C_ 54.6, 96.4, 135.7, 145.6, 169.2, 176.1), and a methoxy group at δ_C_ 51.9 (Figures S14 and S16).

The HMBC correlations from H-5, H-6, H-8, and H-15 to C-7 and from O-CH_3_ and H-15 to C-2 revealed the indolinic moiety. The presence of an *Aspidosperma* skeleton [34] was supported by the HMBC correlations from H-15, H-3, and H-19 to C-21 and from H-18 and H-19 to C-20 (Figure S17). Moreover, the HMBC correlations from O-CH_3_ to C-17 and from H-15 to C-16 indicated the attachment of the methoxy group at C-16 (Figure 2).

The ROEs correlations (Figure 3 and Figure S18) were not sufficient to determine the configuration of the stereogenic carbons C-7, C-15, C-20, and C-21. The absolute configuration of these carbons was determined by comparison of the experimental and calculated ECD data [35]. The experimental spectrum (Figure 4) showed negative Cotton effects (CE) at 211 and 238 nm and positive CE at 201, 259, and 315 nm that matched well with the calculated ECD curve for *7S*, *15R*, *20R*, and *21S*. These configurations are different from those described for tubotaiwine, which were *7S*, *15S*, *20S*, and *21R* [36]. Compound **15** was identified as methyl (*7S,15R,20R,21S*)-20-ethyl-5,6,14,15,21-hexahydro-15,21-ethanopyrrolo[5,21-*d*]carbazole-16-carboxylate-methane and named (*7S,15R,20R,21S*)-tubotaiwine.

### 2.4. Evaluation of the Antiprotozoal Activity

Compounds for which a sufficient amount was available (**1**–**7**, **10**–**13**, and **15**–**16**) were evaluated for their in vitro antiprotozoal activity against *T. b. rhodesiense*, *T. cruzi*, *L. donovani*, and *P. falciparum*, as well as for their cytotoxicity towards L6 cells. The results are summarized in Table 1. Compounds were considered inactive when their IC_50_ values were >50 µM against the parasites, except for compound **12** that could not be tested above 10 µM due to the low amount of compound available. Secoxyloganin (**16**) exhibited antileishmanial activity with an IC_50_ value of 25.3 µM against *L. donovani* and a selectivity index of 11.6. Tubotaiwine (**13**) was the most active compound with an IC_50_ value of 8.5 µM against *P. falciparum* and a selectivity index of 23.4. To the best of our knowledge, this is the first report on the antileishmanial activity of compound **16** and the antiplasmodial activity of compound **13**. In a previous study, secoxyloganin (**16**) was tested against *T. cruzi* and did not show any activity (IC_50_ > 150 µM) [37], which confirms the results obtained here. Moreover, tubotaiwine (**13**) was previously reported in the literature for its antileishmanial activity against *L. infantum* with an IC_50_ value of 17.3 µM, and no activity against *T. cruzi* [38]. Its isomer, (*7S,15R,20R,21S*)-tubotaiwine (**15**), showed no antiprotozoal activity (IC_50_ > 50 µM), and none of the tested compounds showed toxicity towards L6 myoblast cells. A study revealed that the antiplasmodial activity of *Pleiocarpa* spp. was due to the presence of alkaloids [4]. Indeed, five alkaloids isolated from the methanol root extract of *P. mutica* were evaluated against *P. falciparum*, and pleiomutinine showed significant in vitro antiplasmodial activity with an IC_50_ value of 5 µM. Conversely, kopsinine (**2**) and pleiocarpine (**11**) were inactive (IC_50_ > 200 µM). These results are in accordance with our data. Nevertheless, in an in vivo mouse model, compound **11** was found to be moderately active against *P. berghei*, where daily doses of 25 mg/kg/day reduced parasitemia by 28.5% compared to untreated control mice [4].

## 3. Materials and Methods

### 3.1. General Experimental Procedures

Optical rotation measurements were performed using a JASCO P-1030 polarimeter (Easton, MD, USA; methanol, *c* in g/100 mL). The ECD spectra were acquired on a JASCO J-815 CD spectrometer (Easton, MD, USA; methanol). The UV spectra were recorded using a Perkin-Elmer Lambda-25 UV-vis spectrophotometer (Wellesley, MA, USA; methanol). IR spectra were obtained using a Perkin-Elmer Spectrum 100 spectrometer. NMR spectroscopic data were obtained on a Bruker Avance III HD 600 MHz NMR spectrometer equipped with a QCI 5 mm Cryoprobe and a SampleJet automated sample changer (Bruker BioSpin, Rheinstetten, Germany). Chemical shifts (δ) are given in parts per million (ppm) based on the methanol-*d*_4_ signals (δ_H_ 3.31; δ_C_ 49.0) for ^1^H- and ^13^C-NMR experiments, respectively. Coupling constants (*J*) are reported in Hertz (Hz). HRMS data were measured on a Q Exactive Focus Hybrid quadrupole-orbitrap mass spectrometer (Thermo Scientific, Waltham, MA, USA) using electrospray ionization in positive-ion mode. UHPLC-PDA-MS measurements were performed using an Acquity UPLC I-class System (Waters, Milford, MA, USA) equipped with an Acquity PDA detector and a Quattro Micro triple quadrupole mass spectrometer (Waters) using an ESI source operating in positive-ion mode. The separation was performed on a Kinetex EVO C_18_ UPLC column (100 × 2.1 mm i.d., 1.7 μm) (Waters). The flow rate was set to 0.5 mL/min using a gradient (acetonitrile and water both containing 0.1% formic acid) from 5 to 98% acetonitrile in 15 min. The column was then washed with 98% acetonitrile for 2 min and equilibrated with 5% acetonitrile for 2 min. The injection volume was 2 μL and the column temperature set to 40 °C. The UV absorbance was measured at 210 nm, and PDA absorption spectra were recorded between 190 and 500 nm (1.2 nm steps). Fractionation was done on an Armen Spot preparative chromatographic system (Interchim, Montluçon, France) equipped with a quaternary pump, a fraction collector, and a UV detector. Semi-preparative chromatography was carried out on an Armen Spot System (Saint-Avé, France) using a Kinetex Axia Core-Shell C_18_ column (5 μm, 250 × 21.2 mm; Phenomenex, Torrance, CA, USA). Each fraction was analyzed on an Acquity UPLC System (Waters) with an Acquity BEH C_18_ column (50 × 2.1 mm i.d., 1.7 μm) (Waters).

### 3.2. Plant Material

*Pleiocarpa bicarpellata* (Apocynaceae) was collected in Ndjoré at the Centre Region of Cameroon in February 2019 and identified by Victor Nana (Botanist at National Herbarium, Yaoundé, Cameroon). A voucher specimen (N° 30598/HNC) was deposited at the National Herbarium in Yaoundé, Cameroon.

### 3.3. Extraction and Isolation

The roots of *P. bicarpellata* (500 g) were air-dried, crushed into small pieces, and then powdered and extracted with dichloromethane/methanol (1:1) (3 × 5 L, room temperature, 48 h) to afford 4 g of crude extract. The stems of *P. bicarpellata* (800 g) were also dried, crushed into small pieces, powdered, and extracted with methanol (3 × 4 L, room temperature, 48 h) to yield 15 g of crude extract.

The dichloromethane/methanol (1:1) root extract of *P. bicarpellata* (1.9 g) was mixed with 4 g of Celite 577 (Fluka, AG, Buchs, Switzerland) and introduced into a cartridge for a dry load injection. Fractionation was performed using two flash chromatography columns connected in series (PF-C_18_HQ/120 g, 15 µm C_18_, Interchim) with a linear gradient of 5 to 30% acetronitrile containing 0.1% formic acid in 90 min and then up to 100% acetronitrile in 40 min. The flow rate was set to 30 mL/min, and UV detection was performed at 254 nm. The separation yielded 173 fractions that were combined into 38 fractions according to their chromatographic profiles. Fraction 2 directly yielded 10-hydroxy-N_b_-methyl-corynantheol (**1**, 15 mg). Fractions 4, 6, 8, 11, 12, 13, 16, 18, and 20 were selected for further purification using a semi-preparative HPLC with an X-Select C_18_ column (5 µm, 250 × 19.0 mm, Waters). Each fraction was separated using a gradient of 10 to 40% methanol containing 0.1% formic acid in 40 min and then up to 100% methanol in 30 min, except for fractions 6 and 11 that were separated using a gradient of 5 to 20% methanol in 40 min followed by an increase to 100% methanol in 30 min. The flow rate was set to 15 mL/min and UV absorbance was measured at 210 nm. Fraction 4 yielded kopsinine (**2**, 1.2 mg) and secologanoside (**3**, 1.8 mg). Fraction 6 gave loganic acid (**4**, 15 mg). Fraction 8 afforded loganic acid 6′-*O*-β-d-glucopyranosyl (**5**, 3.8 mg). Fraction 11 gave macusine A (**6**, 4.4 mg). N_b_-methyl-corynantheol (**7**, 7 mg) and strictosidinic acid 6′-*O*-β-d-glucopyranoside (**8**, 1.6 mg) were isolated from fraction 12. Dihydro-N_b_-methyl-corynantheol (**9**, 0.7 mg) and strictosidinic acid (**10**, 15 mg) were obtained from fraction 13. Pleiocarpine (**11**, 2.6 mg) and 4-({6-*O*-[(4-hydroxy-3,5-dimethoxyphenyl)carbonyl]-β-glucopyranosyl}oxy)-3,5-dimethoxybenzoic acid (**12**, 0.9 mg) were isolated from fraction 16. Fraction 18 yielded tubotaiwine (**13**, 1.5 mg). Fraction 20 afforded serpentine (**14**, 0.3 mg).

The methanol stem extract of *P. bicarpellata* (5 g) was mixed with 14 g of Celite 577 and introduced into a cartridge for a dry load injection. Fractionation was performed using a flash chromatography column (PF-C_18_HQ/300 g, 15 µm C_18_, Interchim) with a linear gradient of 20 to 25% methanol in 40 min and then up to 100% methanol in 10 min. The flow rate was set to 50 mL/min, and UV detection was performed at 205 nm. The separations yielded 159 fractions that were combined into two fractions, fraction 1 and fraction 2, according to their chromatographic profiles. Fraction 2 (1.7 g) was mixed with 4 g of Celite 577 and introduced into a cartridge for a dry load injection. Fractionation was performed using a flash chromatography column (PF-C_18_HQ/120 g, 15 µm C_18_, Interchim) with a linear gradient of 18 to 25% methanol in 90 min followed by an increase to 100% methanol in 10 min. The flow rate was set to 30 mL/min, and UV detection was performed at 205 nm. The separation yielded 255 fractions that were combined into eight fractions according to their chromatographic profiles (fractions 21–28). Fraction 27 was selected for separation using two flash chromatography columns in series (PF-C_18_HQ/120 g, 15 µm C_18_, Interchim) with a linear gradient of 20 to 40% methanol in 100 min and then up to 100% methanol in 10 min. The flow rate was set to 30 mL/min, and UV detection was performed at 205 nm. The separation yielded 175 fractions that were combined into five fractions according to their chromatographic profiles (fractions 271–275). Fraction 272 was selected for further purification using a semi-preparative HPLC with an X-Select C_18_ column (5 µm, 250 × 19.0 mm, Waters) using a linear gradient of 20 to 40% methanol in 70 min followed by an increase to 100% methanol in 30 min. The flow rate was set to 20 mL/min and UV absorbance was measured at 225 nm. This yielded (*7S,15R,20R,21S*)-tubotaiwine (**15**, 1.2 mg) and secoxyloganin (**16**, 2.0 mg).

### 3.4. Spectral and Physical Data of Compounds ***7*** and ***15***

#### 3.4.1. N_b_-Methyl-Corynantheol (**7**)

Brown oil; ${\left[\alpha \right]}_{D}^{25}\;$+ 4.6 (*c* 0.1, methanol); UV (methanol) *λ*_max_ (log *ε*) 220 (3.04), 273 (2.35), 289 (2.21) nm; IR ν_max_. 3308, 2943, 2832, 1449, 1023 cm^−1^; ^1^H- and ^13^C-NMR, see Table 2; HRESIMS *m*/*z* 311.2114 ([M])^+^ (calcd for C_20_H_27_N_2_O^+^, 311.2118).

#### 3.4.2. (*7S,15R,20R,21S*)-Tubotaiwine (**15**)

Colorless amorphous solid; ${\left[\alpha \right]}_{D}^{25}\;$+ 460.3 (*c* 0.07, methanol); UV (methanol) *λ*_max_ (log *ε*) 220 (3.11), 296 (2.85), 336 (2.89) nm; ^1^H- and ^13^C-NMR, see Table 2; HRESIMS *m*/*z* 325.1901 ([M + H])^+^ (calcd for C_20_H_24_N_2_O_2_, 325.1911). 

### 3.5. MS Data Treatment, Molecular Network Generation, and Annotation

Thermo .RAW files were converted into .mzXML (mass spectrometry data format) using MSConvert software.36, part of the Proteowizard package (ProteoWizard, Palo Alto, CA, USA) [39]. The converted files were uploaded to MZmine software suite version 2.53 [16]. For mass detection at MS^1^ level, the noise level was set to 1.0 × 10^6^. For MS^2^ detection, the noise level was set to 0.00. The ADAP chromatogram builder parameters were set as follows: minimum group size of scans, 5; minimum group intensity threshold, 1.0 × 10^6^; minimum highest intensity, 1.0 × 10^6^ and *m*/*z* tolerance of 8.0 ppm. The ADAP algorithm (wavelets) was used for chromatogram deconvolution with the following parameters: S/N tolerance, 25; minimum feature height, 1.0 × 10^6^; coefficient area threshold, 100; peak duration range, 0.02–1.0 min; RT wavelet range, 0.02–0.08 min. Isotopes were detected using the isotope peak grouper with a *m*/*z* tolerance of 8.0 ppm, a RT tolerance of 0.02 min (absolute), the maximum charge set at 1, and the representative isotope used was the most intense. Each file was filtered by retention time within a range from 0.70 to 8.00 min, and only the ions with an associated MS^2^ spectrum were kept. Alignment was done with the join-aligner (*m*/*z* tolerance, 8.0 ppm; RT tolerance, 0.05 min) comparing the spectral similarity (spectral tolerance, 8.0 ppm; MS level, 2; Weighted dot-product cosine with default parameters). The resulting aligned peak list was exported for further analysis as a .mgf file. 

The .mgf file was exported from MZmine to build the molecular network, using the online workflow (https://ccms-ucsd.github.io/GNPSDocumentation/) on the GNPS website (http://gnps.ucsd.edu). The data were clustered with the following parameters: precursor ion mass tolerance: 0.02 Da; MS/MS fragment ion tolerance: 0.02 Da; minimum cosine score: 0.7; and minimum matched peaks: 6. The spectra in the network were then searched against the spectral libraries of GNPS. The library spectra were filtered in the same manner as the input data. The required library matches were set to show a score above 0.6 and at least 3 matched peaks. The job can be found here: https://gnps.ucsd.edu/ProteoSAFe/status.jsp?task=56ea2e86598848a5b6750a445be60c74. The output of the GNPS platform was compared against an in silico database to extend the rate of putative annotations [40]. This output was subjected to taxonomically informed metabolite annotation [19] to re-rank and clean up the output based on the taxonomy of the collection. The in silico database used for all this process includes the combined records of the Dictionary of Natural Products (https://dnp.chemnetbase.com/) and Lotus (https://lotus.naturalproducts.net/) [41].

### 3.6. ECD Computational Details

The absolute configuration of compounds **7** and **15** was assigned according to the comparison of the calculated and experimental ECD spectra. Based on the structure proposed by NMR experiments, conformers were generated using the MMFF94s force field with Spartan Student v7 (Wavefunction, Irvine, CA, USA). From the results obtained, the 10 isomers with the lowest energy were subjected to further successive PM3 and B3LYP/6-31G(d,p) optimizations with Gaussian 16 software (Gaussian Inc., Wallingford, CT, USA) using the CPCM model in methanol. All optimized conformer outputs were checked to avoid imaginary frequencies after each optimization. A cut-off of 4 kcal/mol was set as maximum difference between conformers. The remaining conformers were submitted to Gaussian 16 software for ECD calculations, using B3LYP/def2svp as basis set with the CPCM model in methanol. The computation in Gaussian was performed at the University of Geneva on the Baobab cluster (https://plone.unige.ch/distic/pub/hpc/baobab_en). The calculated ECD spectra were generated in SpecDis1.71 software (Berlin, Germany) based on a Boltzmann weighing average.

### 3.7. Antitrypanosomal, Antileishmanial, Antiplasmodial, and Cytotoxicity Assays

The in vitro activity was assessed on *T. b. rhodesiense* (STIB900, bloodstream forms), *T. cruzi* (Tulahuen C2C4, intracellular amastigotes), *L. donovani* (MHOM-ET-67/L82, axenically grown amastigotes), *P. falciparum* (NF54, intraerythrocytic), and L6 cells (rat skeletal myoblasts) as previously described [42]. Results are expressed in µg/mL for extracts and in µM for pure compounds. Samples were considered active when their IC_50_ values were <50 µg/mL for extracts and <50 µM for pure compounds.

## 4. Conclusions

To date, this is the first description of the chemical constituents, antiprotozoal activity, and dereplication of *P. bicarpellata* extracts. This study led to the isolation of two new alkaloids and fourteen known compounds, including six indole alkaloids, four indoline alkaloids, one secoiridoid glycoside, two iridoid glycosides, and one phenolic glucoside (**1**–**16**). Compounds **3**–**6**, **8**, **10**, **12**, and **14** are reported here for the first time in the *Pleiocarpa* genus. Among the tested compounds, only tubotaiwine (**13**) showed significant activity and selectivity against *P. falciparum*.

## Figures and Tables

**Table 1:** Antiparasitic activity and cytotoxicity of extracts and compounds from *P. bicarpellata*.

Sample	Plant Extract	IC_50_ ^a^ (µM)			SI ^c^
		*L. donovani*	*P. falciparum*	Cytotoxicity ^b^	
Root	Methanol: dichloromethane	>50	3.5 ^d^	>100 ^d^	
Stem	Methanol	>50	34.2 ^d^	>100 ^d^	
**6**		>50	26.9	272.1	10.1
**11**		>50	26.6	155.5	5.8
**13**		>50	8.5	197.4	23.2
**16**		25.3	>50	293.7	11.6
Miltefosine		0.554			
Chloroquine			0.006		
Podophyllotoxin				0.027	

^a^ The IC_50_ are the means of two independent assays. ^b^ Rat skeletal myoblast (L6 cells). ^c^ Selectivity index (SI) = IC_50_ cytotoxicity/IC_50_ against parasite. ^d^ IC_50_ values expressed in µg/mL.

**Table 2:** ^1^H-NMR (DMSO-*d*_6_, 600 MHz) and ^13^C-NMR (DMSO-*d*_6_, 150 MHz) data of compounds **7** and **15**.

Position	7		15	
	δ_H_ (*J* in Hz)	δ_C_, Type	δ_H_ (*J* in Hz)	δ_C_, Type
2		128.5, C		176.1, C
3	4.66, m	65.9, CH	2.05, m 2.95, m	42.9, CH_2_
5	3.49, sept (3.6)	59.5, CH_2_	3.23, s 3.50, s	53.5, CH_2_
6	3.13, m 3.23, m	18.0, CH_2_	3.00, m 3.38, m	46.6, CH_2_
7		105.4, C		54.6, C
8		126.8, C		135.7, C
9	7.51, d (7.7)	119.1, CH	7.34, d (7.5)	120.8, CH
10	7.08, t (7.1)	120.7, CH	6.93, td (7.4)	122.6, CH
11	7.18, t (7.1)	123.5, CH	7.18, td (7.7)	129.6, CH
12	7.38, d (7.9)	112.4, CH	6.98, d (7.9)	111.7, CH
13		138.3, C		145.6, C
14	2.28, m 2.65, m	30.9, CH_2_	1.95, m	27.9, CH_2_
15	3.23, m	30.4, CH	3.22, s	31.2, CH
16	1.42, h 1.60, h	35.4, CH_2_		96.4, C
17	3.79 (m) 3.86 (m)	59.6, CH_2_		169.2, C
18	1.82, d (6.8)	13.4, CH_3_	0.75, t (7.2)	11.4, CH_3_
19	5.97, q (6.9)	132.0, CH	0.92, m	24.3, CH_2_
20		129.0, C	2.05, m	41.3, CH
21	3.69, d (12.7) 4.35, d (12.8)	63.8, CH_2_	4.40, s	66.6, CH
NCH_3_	3.18, s	48.8		
OCH_3_			3.79, s	51.9, CH_3_
NH			8.57, s	

## Data Availability

The data are archived at the authors’ institution.

## Supplementary Materials

The following supporting information can be downloaded at: https://www.mdpi.com/article/10.3390/molecules27072200/s1, Figure S1: Molecular network from *Pleiocarpa bicarpellata* extracts; Figure S2: IR spectrum of **7**; Figure S3: UV spectrum of **7**; Figure S4: HRESIMS spectrum of **7**; Figure S5: ^1^H-NMR spectrum of **7**; Figure S6: DEPTQ NMR spectrum of **7**; Figure S7: COSY NMR spectrum of **7**; Figure S8: HSQC NMR spectrum of **7**; Figure S9: HMBC NMR spectrum of **7**; Figure S10: ROESY NMR spectrum of **7**; Figure S11: UV spectrum of **15**; Figure S12: HRESIMS spectrum of **15**; Figure S13: ^1^H-NMR spectrum of **15**; Figure S14: DEPTQ NMR spectrum of **15**; Figure S15: COSY NMR spectrum of **15**; Figure S16: HSQC NMR spectrum of **15**; Figure S17: HMBC NMR spectrum of **15**; Figure S18: ROESY NMR spectrum of **15**.

## Sample Availability

Samples of the compounds are available from the authors upon request.
