# Supplementary material for: Isolation and Structural Elucidation of Compounds from Pleiocarpa bicarpellata and Their In Vitro Antiprotozoal Activity
Source: Molecules. 2022 Mar 28;27(7):2200. doi: 10.3390/molecules27072200 (PMC9000413; doi:10.3390/molecules27072200)
Supplement: Supplementary file 1 [file molecules-27-02200-s001.zip › molecules-1636750-supplementary.pdf]

Supplementary Material

Isolation and Structural Elucidation of Compounds from *Pleiocarpa bicarpellata* and Their In Vitro Antiprotozoal Activity

Ozlem Sevik Kilicaslan [1,2], Sylvian Cretton [1,2], Luis Quirós-Guerrero [1,2], Merveilles A. Bella [3], Marcel Kaiser [4,5], Pascal Mäser [4,5], Joseph T. Ndongo [3] and Muriel Cuendet [1,2,*]

[1] School of Pharmaceutical Sciences, University of Geneva, 1211 Geneva, Switzerland; ozlem.sevik@unige.ch (O.S.K.); sylvian.cretton@unige.ch (S.C.); luis.guerrero@unige.ch (L.Q.-G.)
[2] Institute of Pharmaceutical Sciences of Western Switzerland, University of Geneva, 1211 Geneva, Switzerland
[3] Department of Chemistry, Higher Teacher Training College, University of Yaoundé 1, Yaoundé P.O. Box 47, Cameroon; bella_aurore@yahoo.fr (M.A.B.); thierry.ndongo@ens.cm (J.T.N.)
[4] Swiss Tropical and Public Health Institute, 4002 Basel, Switzerland; marcel.kaiser@swisstph.ch (M.K.); pascal.maeser@swisstph.ch (P.M.)
[5] University of Basel, 4003 Basel, Switzerland
* Correspondence: muriel.cuendet@unige.ch

Table of content

Figure S1. Molecular network from *Pleiocarpa bicarpellata* extracts. Nodes with yellow circles represent ions dereplicated by GNPS and ISDB, and identified as alkaloids. 3
Figure S2. IR spectrum of **7**. 3
Figure S3. UV spectrum of **7**. 4
Figure S4. HRESIMS spectrum of **7** (Q Exactive Focus Hybrid quadripole-orbitrap mass spectrometer, positive mode). 4
Figure S5. ^{1}H NMR (600 MHz, CD_3OD) spectrum of **7**. 5
Figure S6. DEPTQ NMR (150 MHz, CD_3OD) spectrum of **7**. 5
Figure S7. COSY (600 MHz, CD_3OD) spectrum of **7**. 6
Figure S8. HSQC (600 MHz, CD_3OD) spectrum of **7**. 6
Figure S9. HMBC (600 MHz, CD_3OD) spectrum of **7**. 7
Figure S10. ROESY (600 MHz, CD_3OD) spectrum of **7**. 7
Figure S11. UV spectrum of **15**. 8
Figure S12. HRESIMS spectrum of **15** (Q Exactive Focus Hybrid quadripole-orbitrap mass spectrometer, positive mode). 8
Figure S13. ^{1}H NMR (600 MHz, CD_3OD) spectrum of **15**. 9
Figure S14. DEPTQ NMR (150 MHz, CD_3OD) spectrum of **15**. 9
Figure S15. COSY (600 MHz, CD_3OD) spectrum of **15**. 10
Figure S16. HSQC (600 MHz, CD_3OD) spectrum of **15**. 10
Figure S17. HMBC (600 MHz, CD_3OD) spectrum of **15**. 11
Figure S18. ROESY (600 MHz, CD_3OD) spectrum of **15** 11

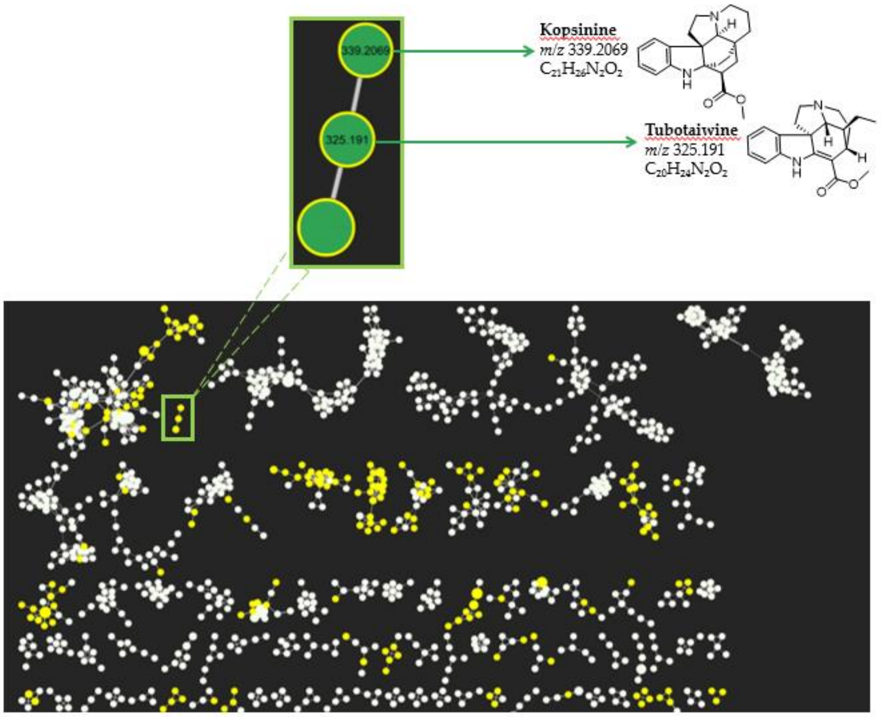

Figure S1. Molecular network from *Pleiocarpa bicarpellata* extracts. Nodes with yellow circles represent ions dereplicated by GNPS and ISDB, and identified as alkaloids.

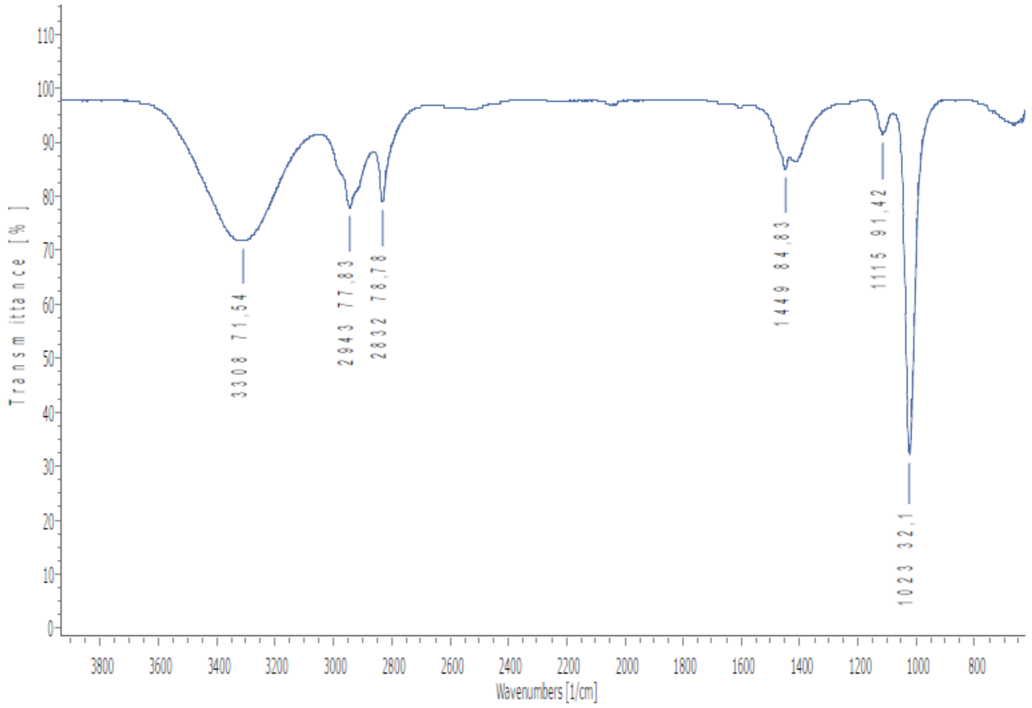

Figure S2. IR spectrum of **7**.

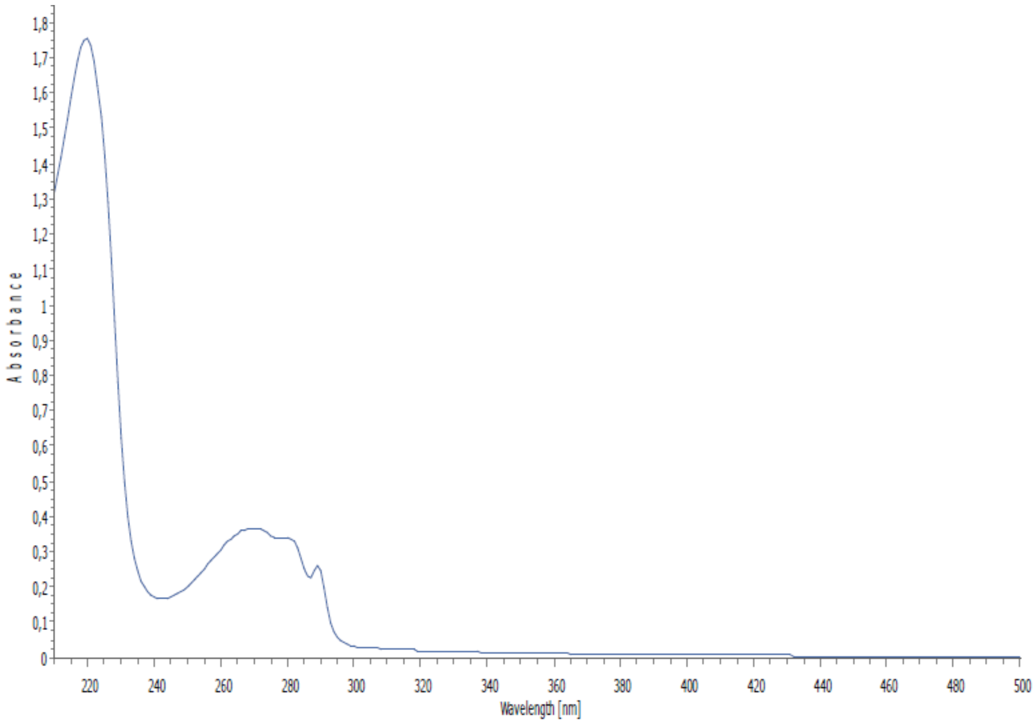

Figure S3. UV spectrum of **7**.

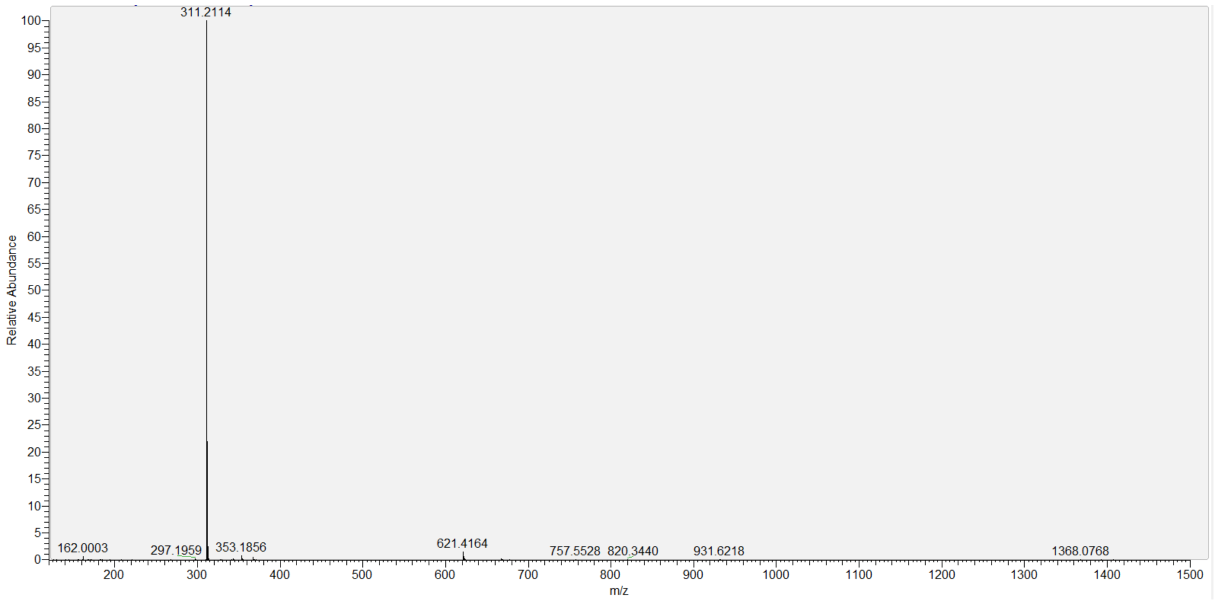

Figure S4. HRESIMS spectrum of **7** (Q Exactive Focus Hybrid quadripole-orbitrap mass spectrometer, positive mode).

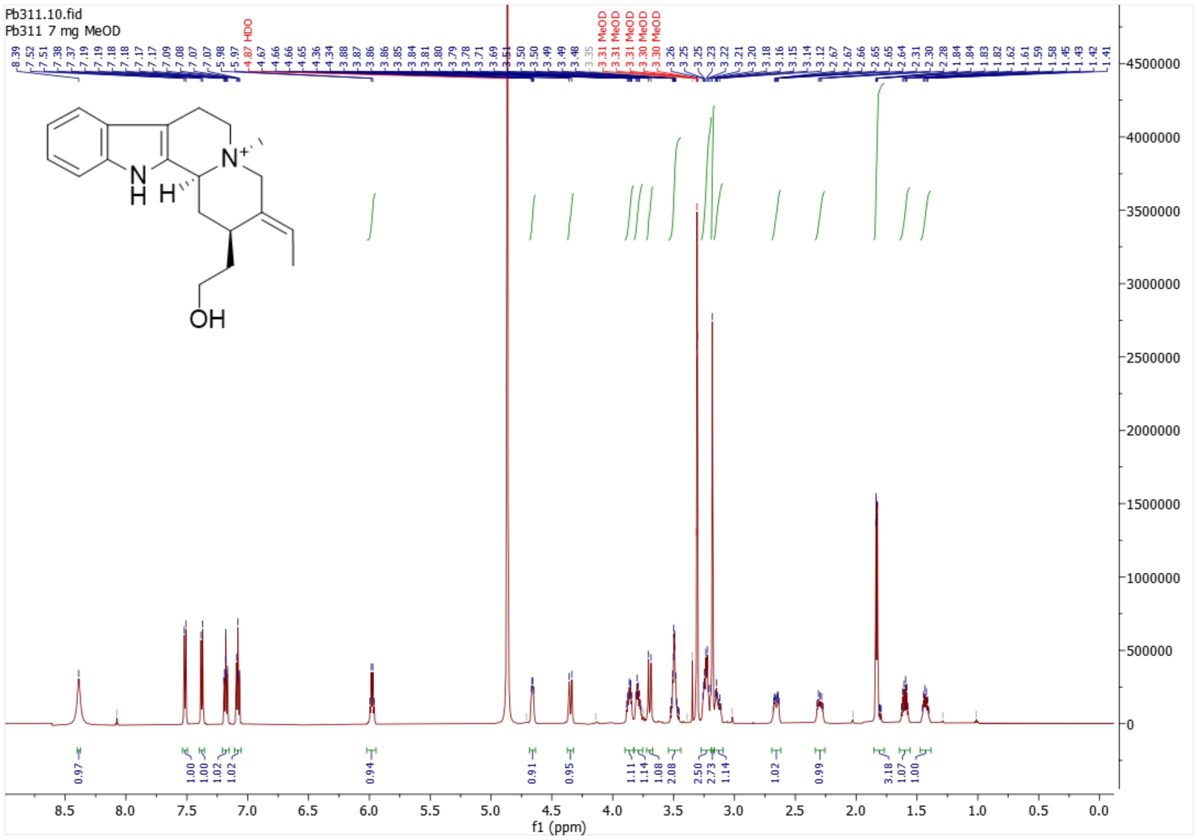

Figure S5. ^{1}H NMR (600 MHz, CD_3OD) spectrum of **7**.

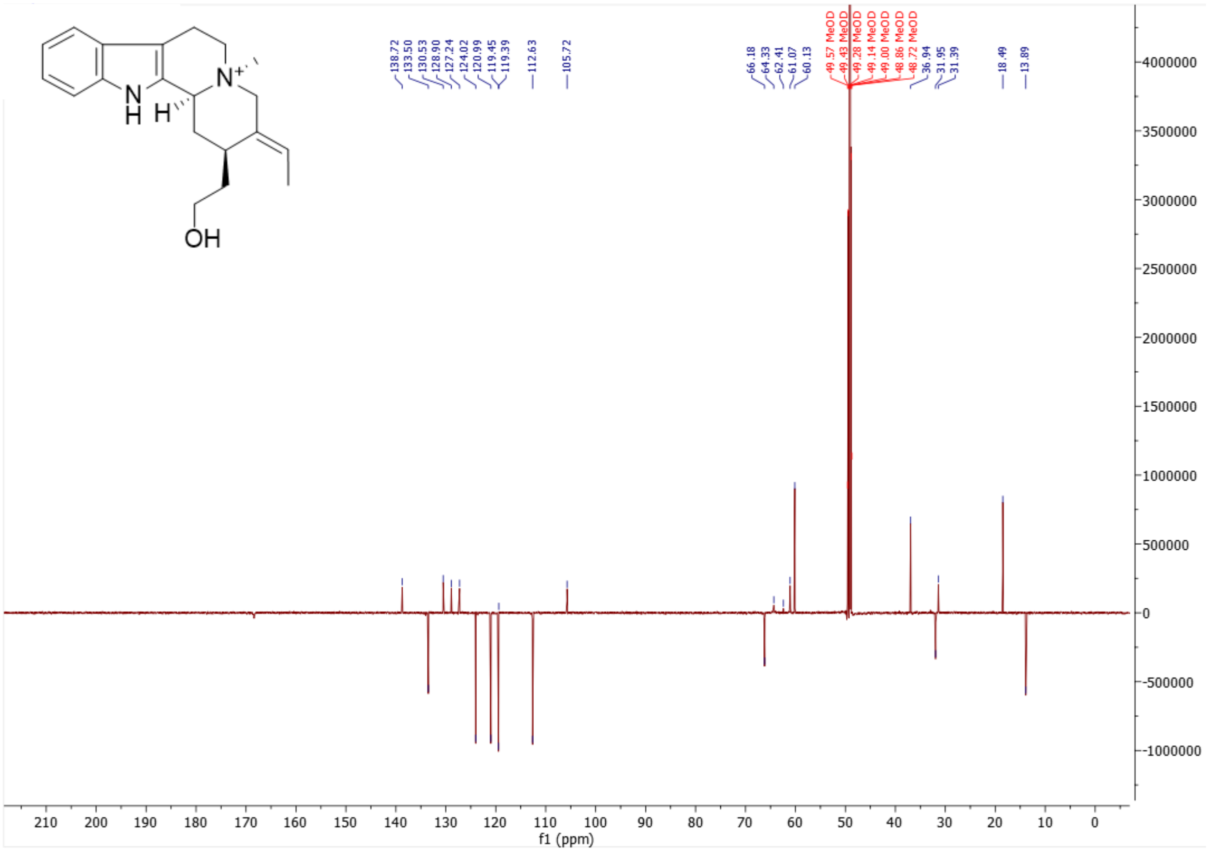

Figure S6. DEPTQ NMR (150 MHz, CD_3OD) spectrum of **7**.

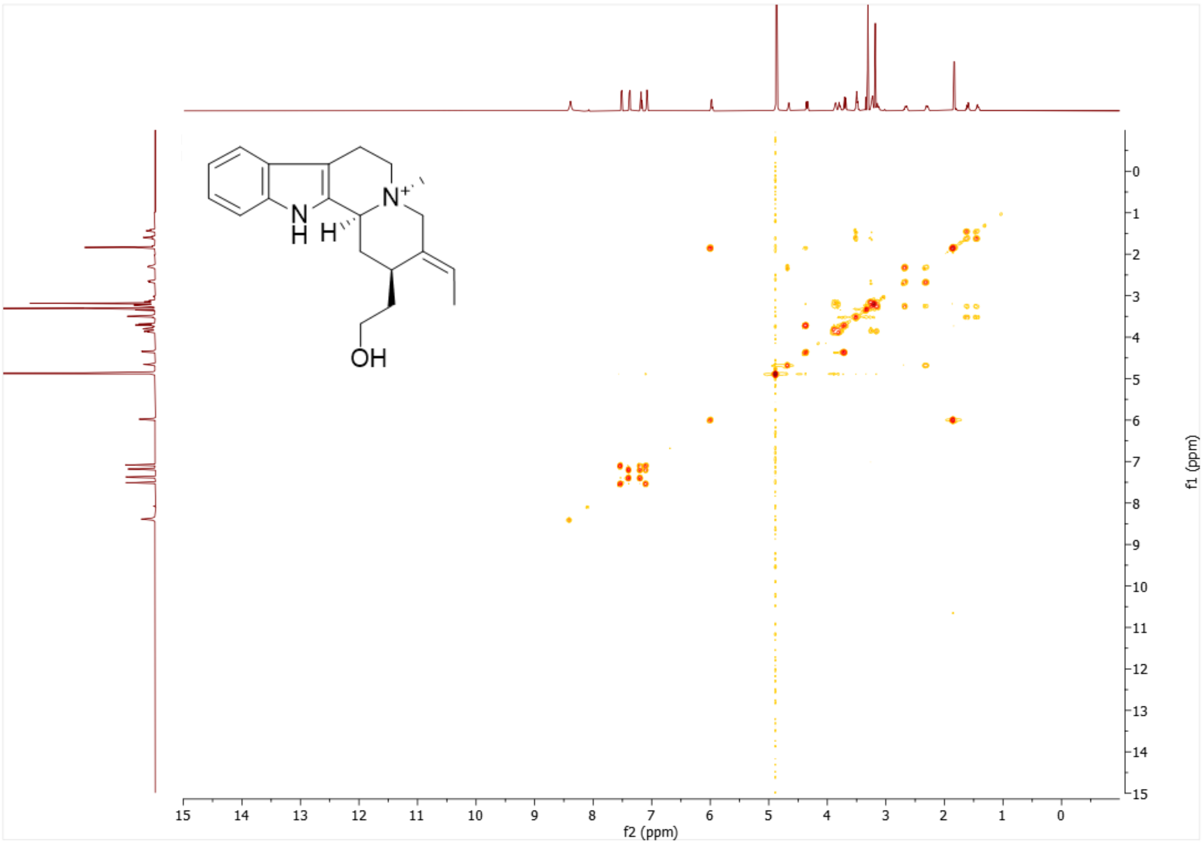

Figure S7. COSY (600 MHz, CD_3OD) spectrum of **7**.

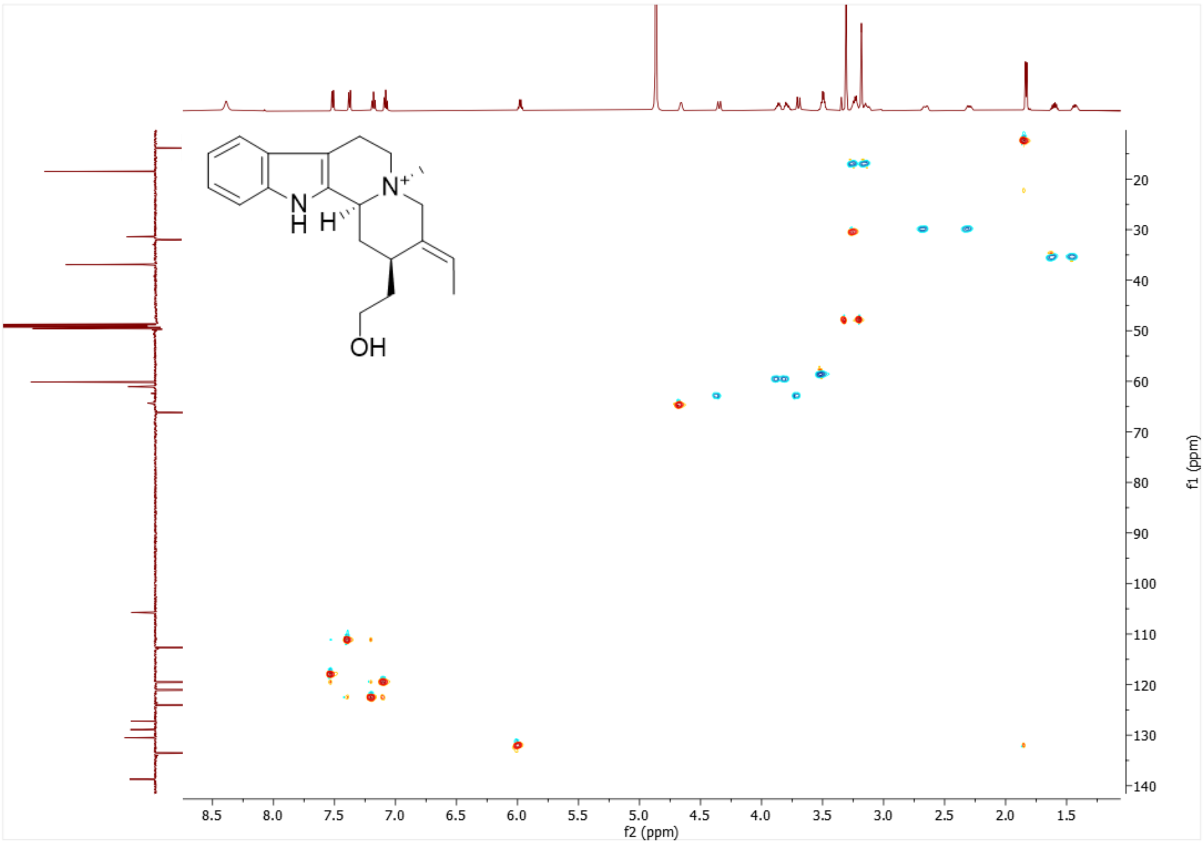

Figure S8. HSQC (600 MHz, CD_3OD) spectrum of **7**.

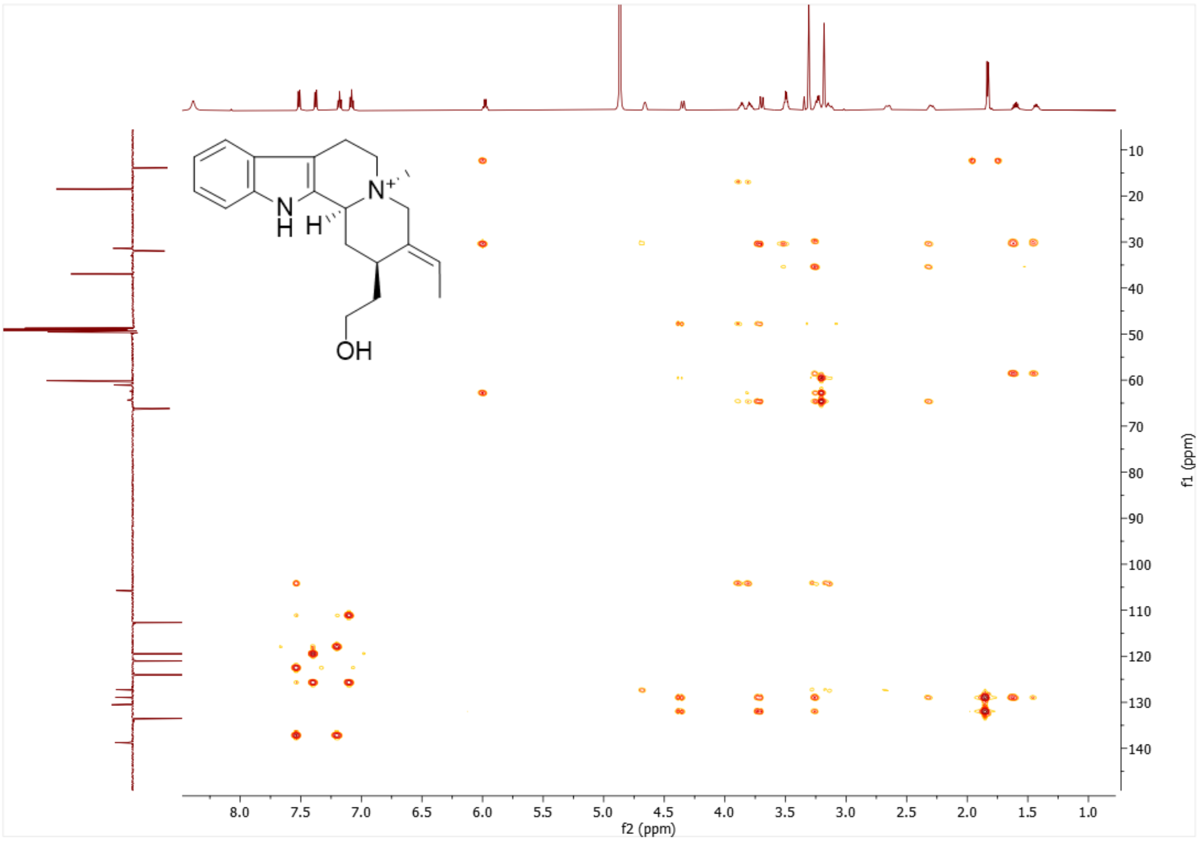

Figure S9. HMBC (600 MHz, CD_3OD) spectrum of **7**.

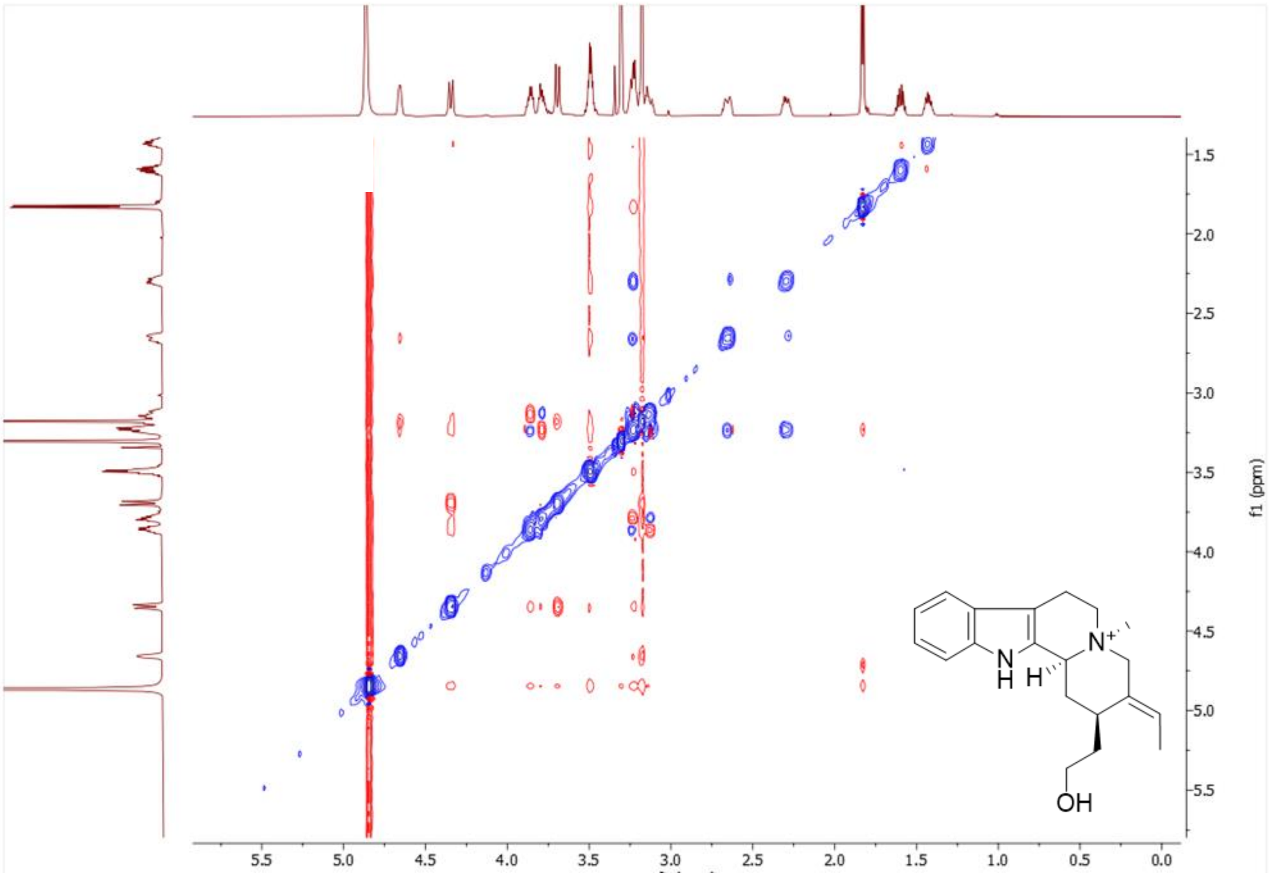

Figure S10. ROESY (600 MHz, CD_3OD) spectrum of 7.

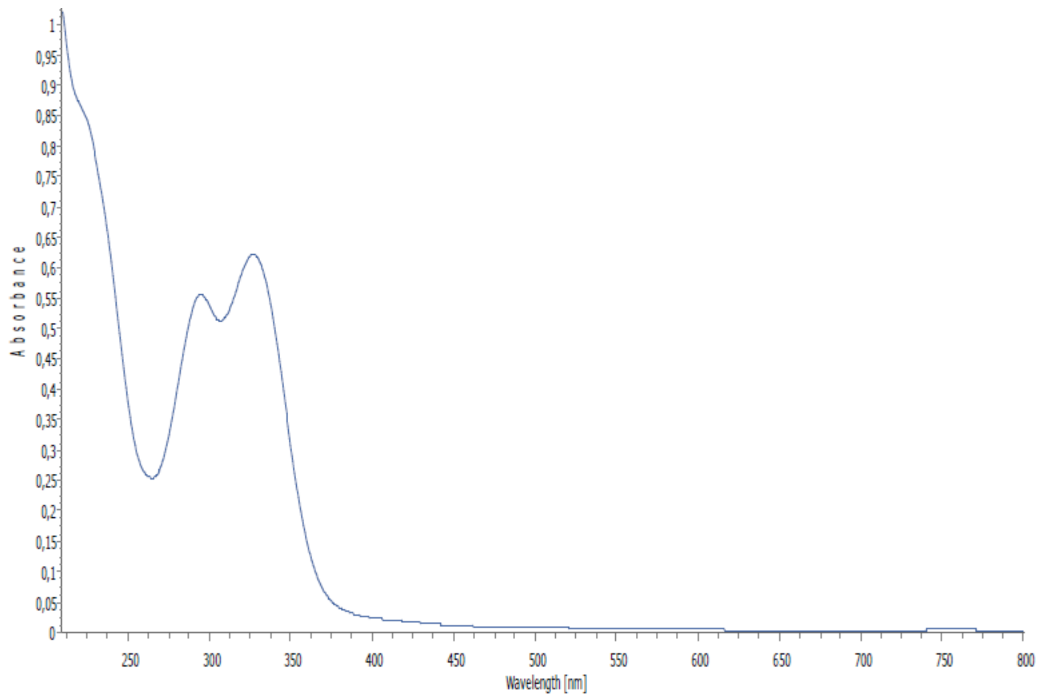

Figure S11. UV spectrum of **15**.

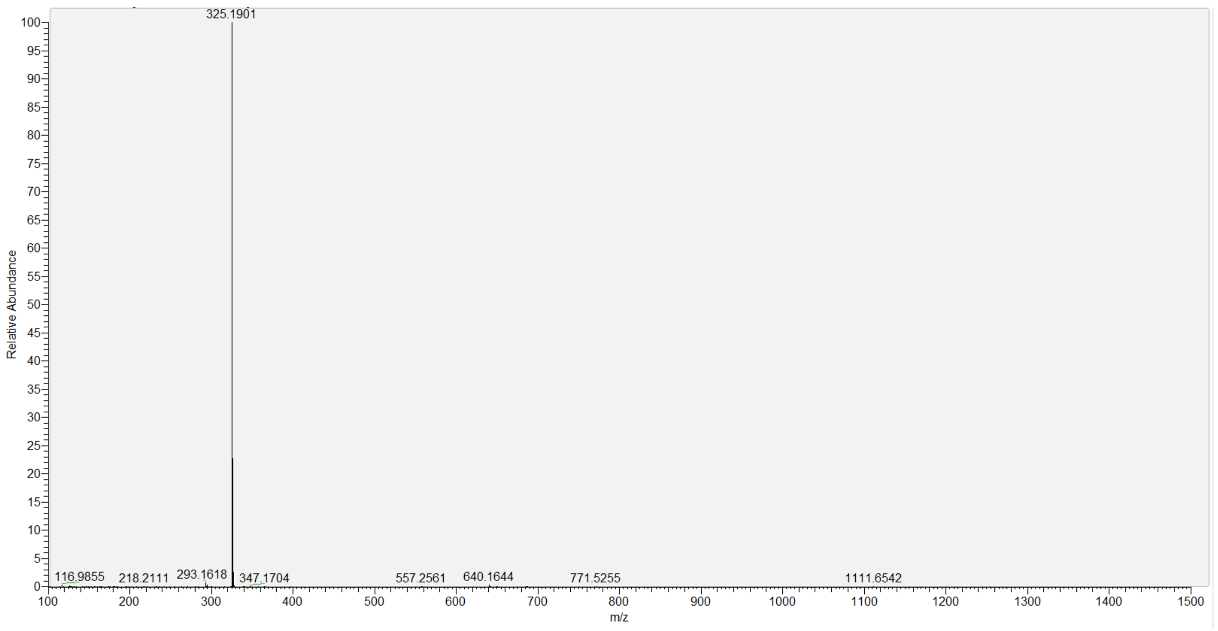

Figure S12. HRESIMS spectrum of **15** (Q Exactive Focus Hybrid quadripole-orbitrap mass spectrometer, positive mode).

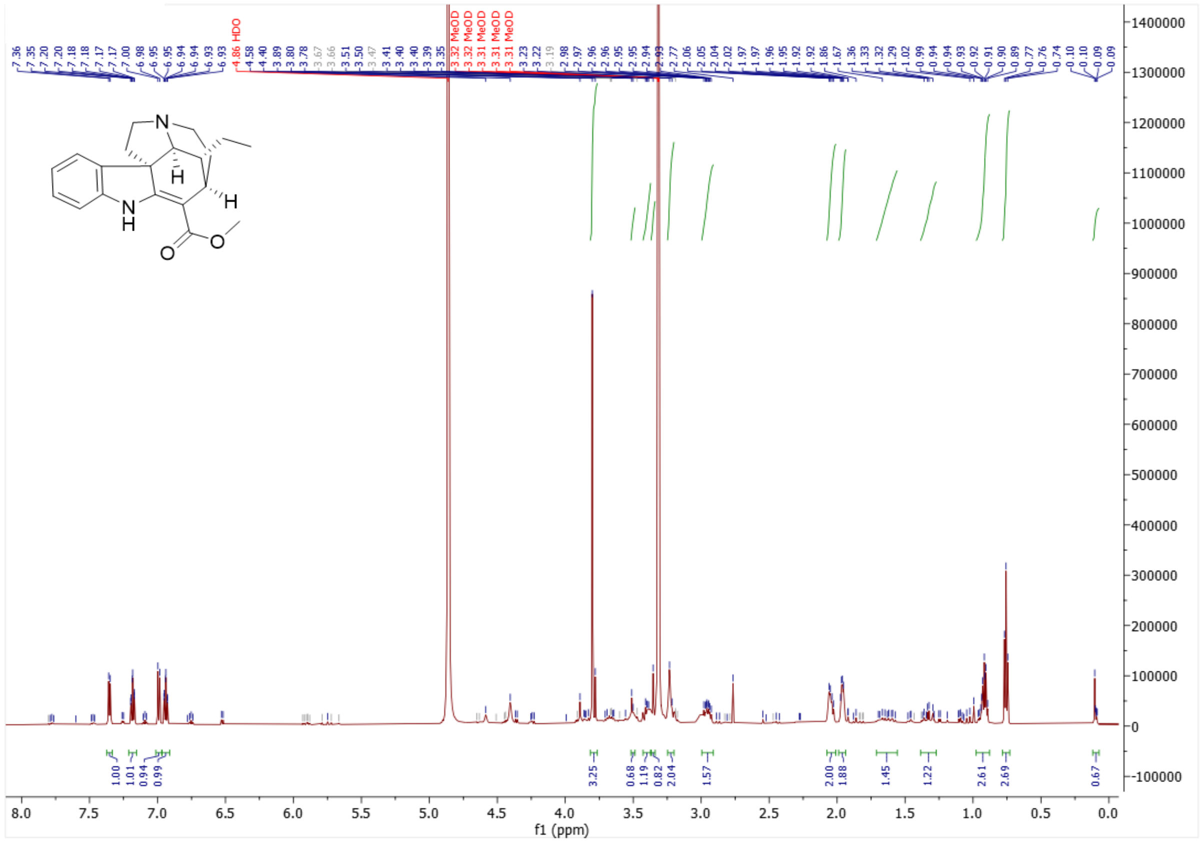

Figure S13. ^{1}H NMR (600 MHz, CD_3OD) spectrum of **15**.

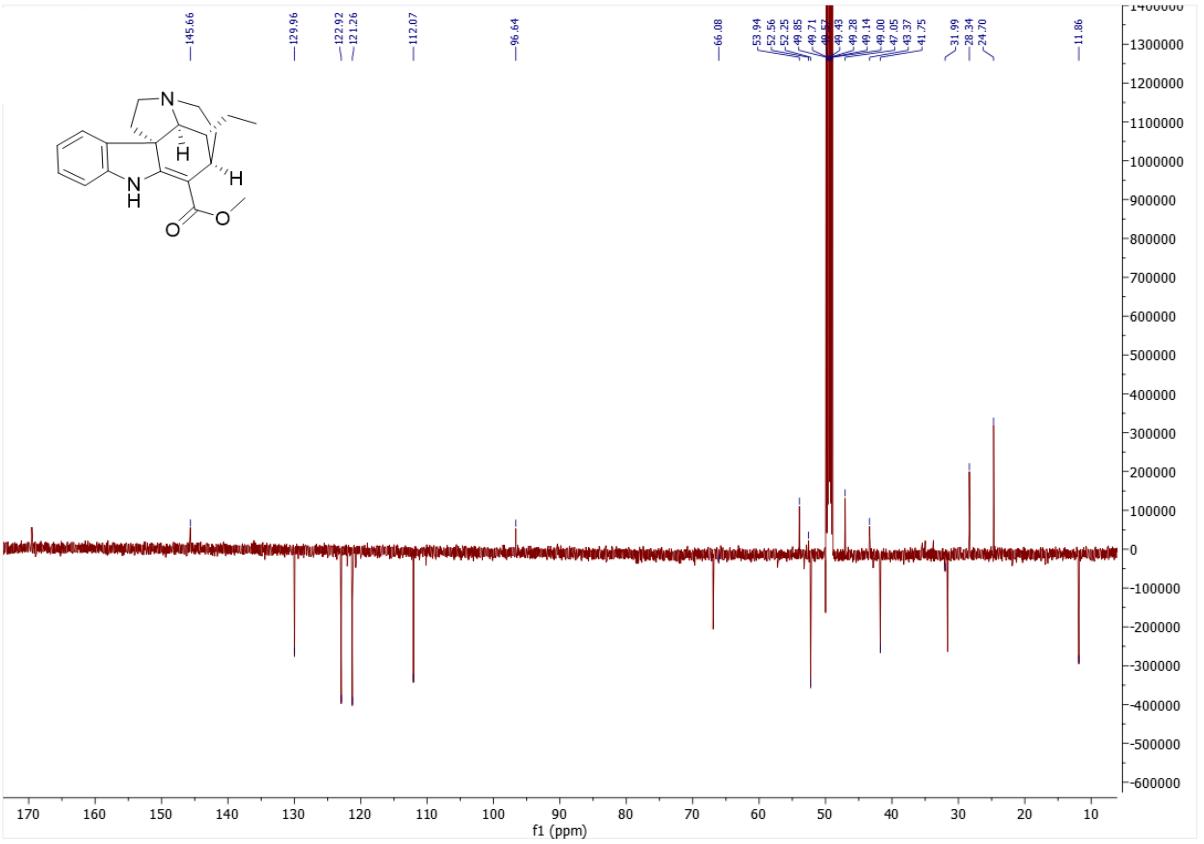

Figure S14. DEPTQ NMR (150 MHz, CD_3OD) spectrum of **15**.

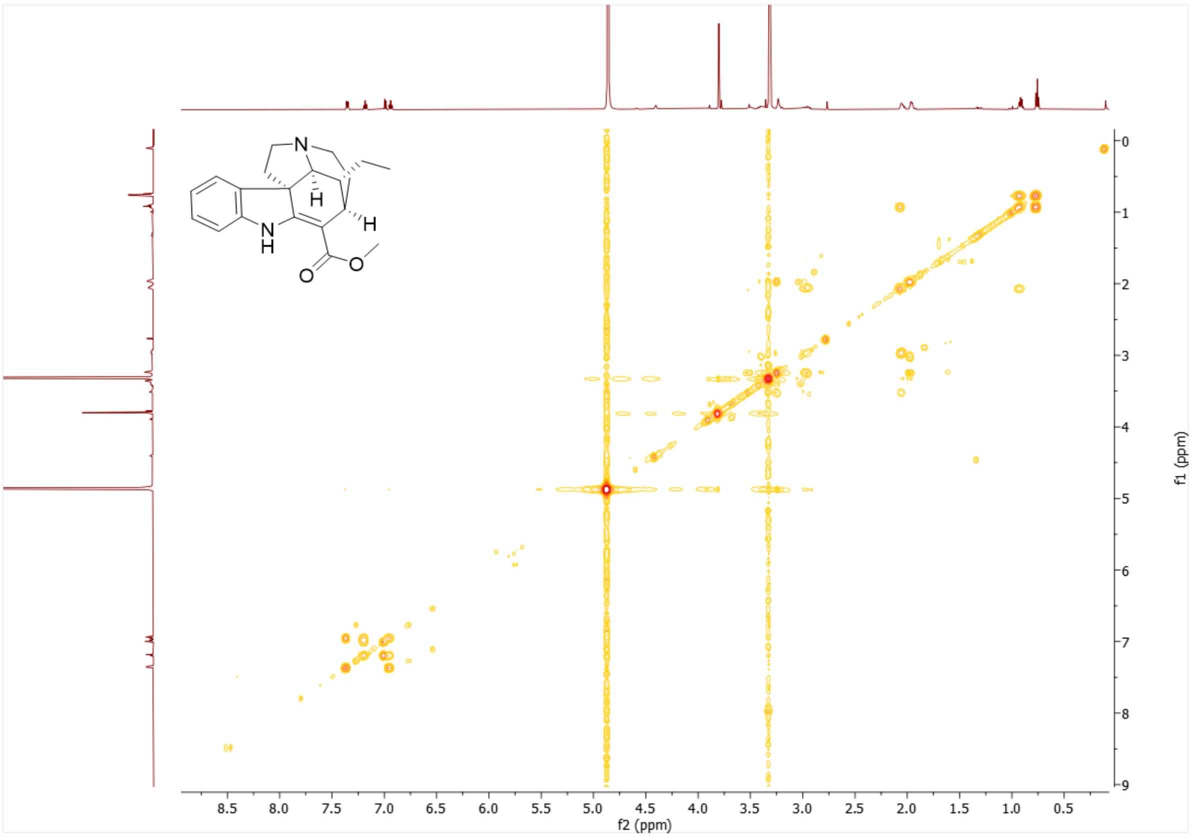

Figure S15. COSY (600 MHz, CD_3OD) spectrum of **15**.

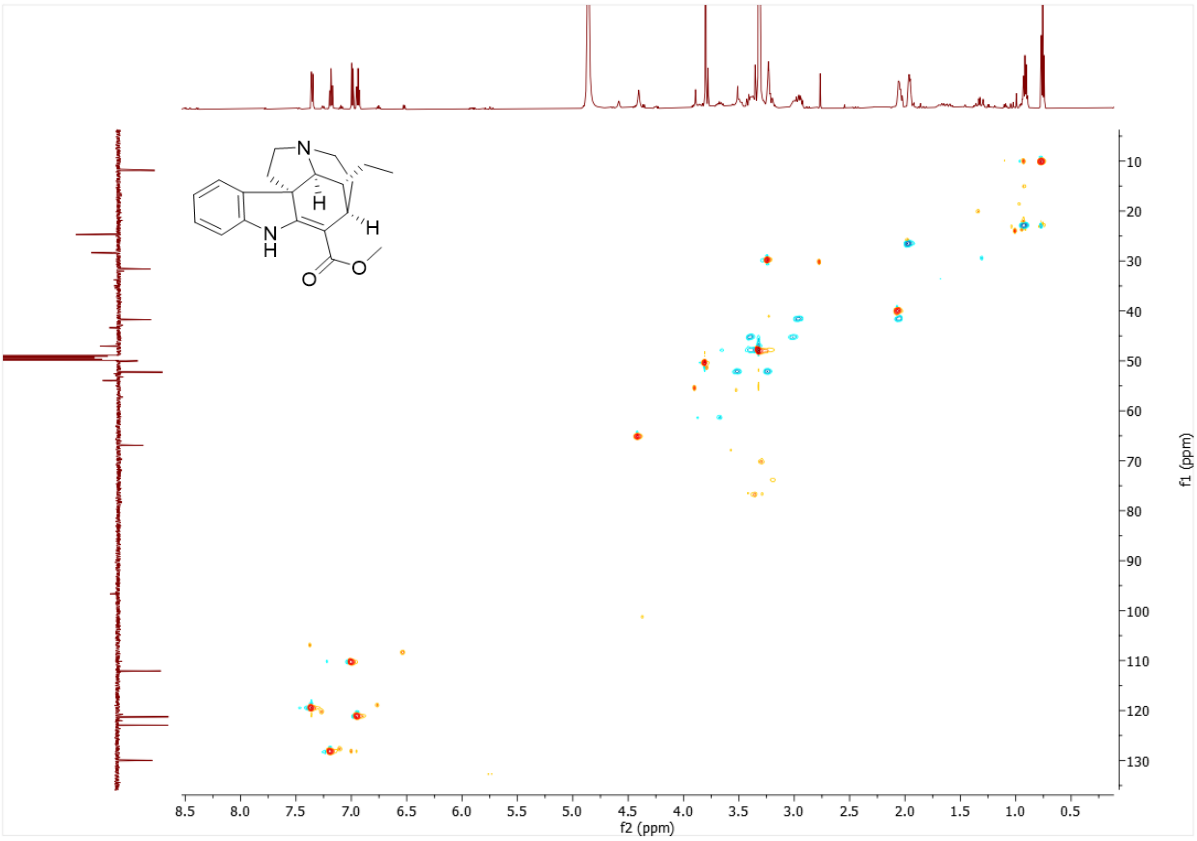

Figure S16. HSQC (600 MHz, CD_3OD) spectrum of **15**.

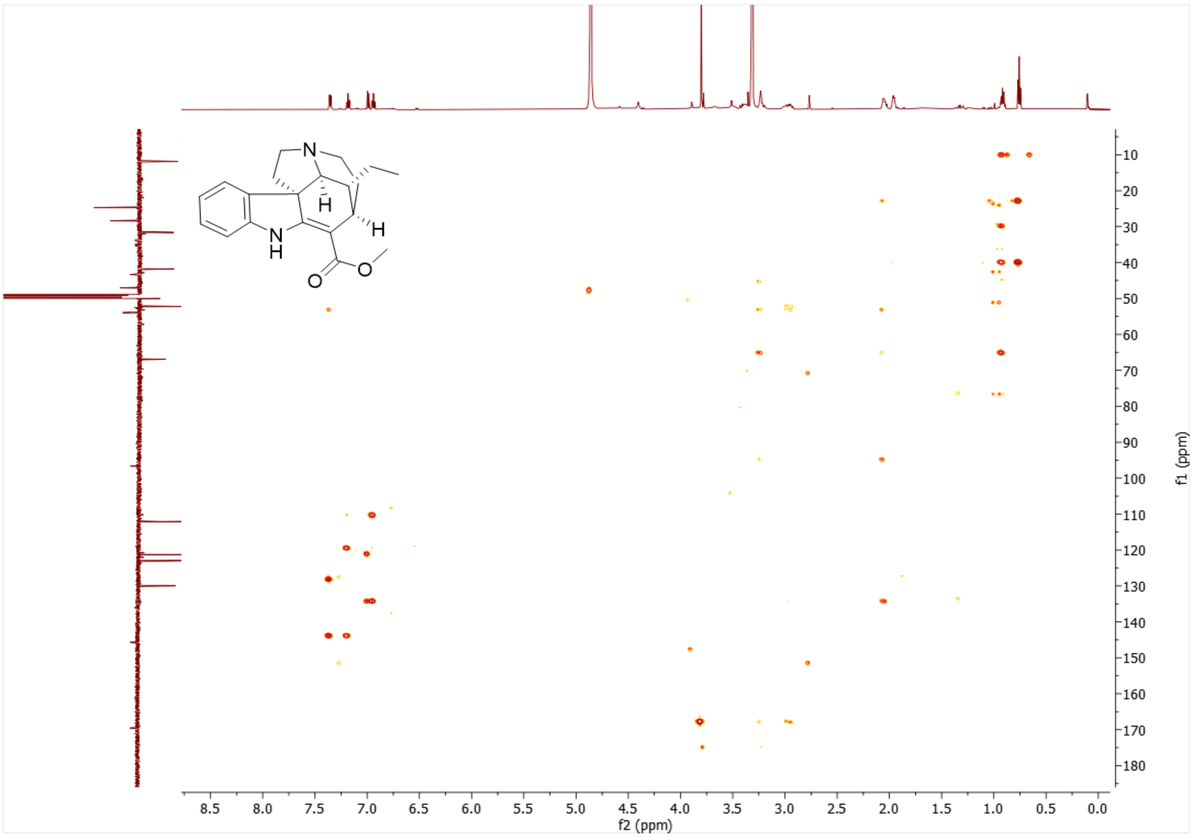

Figure S17. HMBC (600 MHz, CD_3OD) spectrum of **15**.

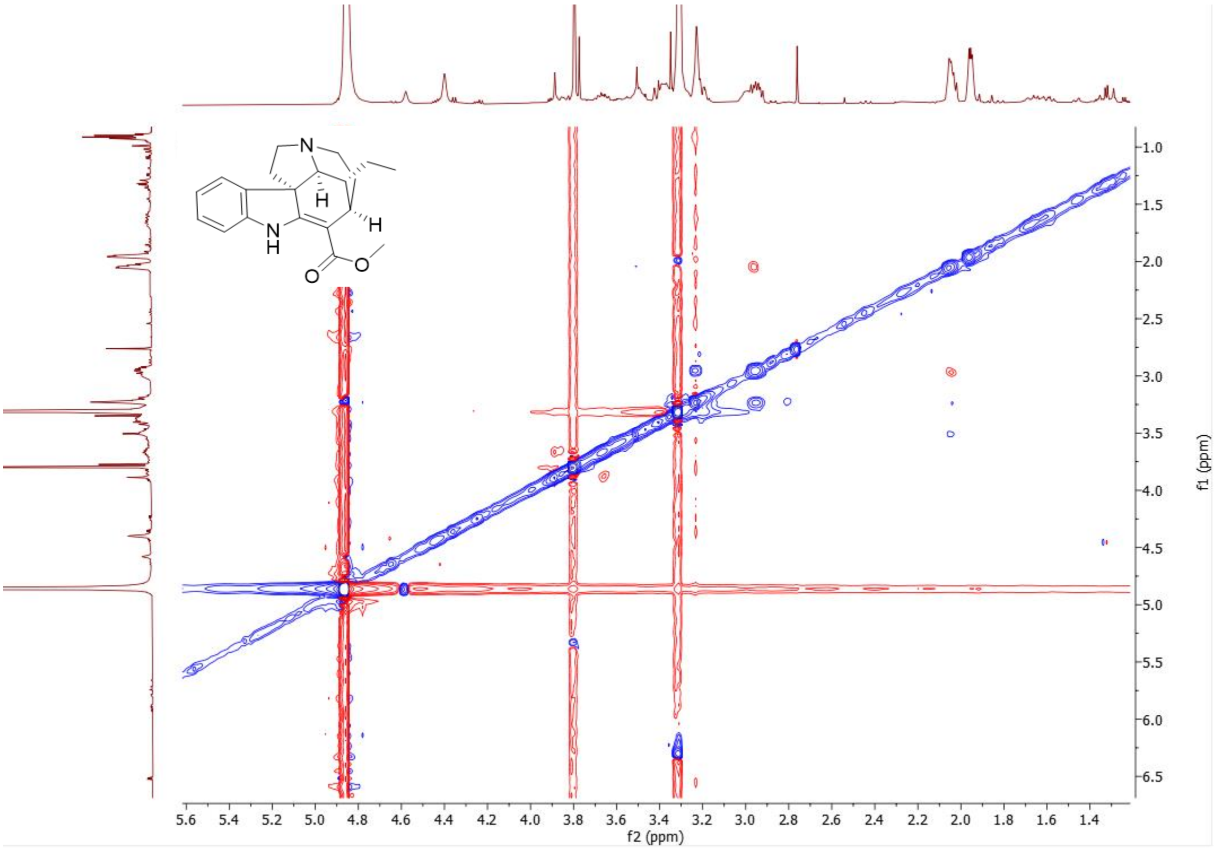

Figure S18. ROESY (600 MHz, CD_3OD) spectrum of **15**
